# New College at Indianapolis

**Published:** 1897-09

**Authors:** 


### New College at Indianapolis.

The first announcement of the Central College of Dentistry, located at Indianapolis, Ind., is just at hand. The propriety of organizing a new dental college is seriously questioned by some. If all the dental colleges that have been established were what they ought to be, or could be, there would be some ground for criticism. There can certainly be no just reason for the establishment of more second, third and fourth-rate schools. Were these just mentioned eliminated, there would be room for several more first-class colleges—colleges thoroughly equipped and rightly conducted, and these are really needed. Whether they shall be started *de novo*, or whether some that are already in existence, shall be reorganized and brought up to the level of the best, is a question about which there may reasonably be some difference of opinion. It is not easy always to elevate an institution that has for a long time been operated upon a low plane, nor is it always easy to start a new enterprise with the best requirements, and make it from the start a financial success, and especially if it has not the fostering care of some well-established, educational institution. The college above referred to is in its beginning state, at least so far as educational work is concerned, though its organization has been completed, the announcement issued, a building has been secured and is being equipped, and the first session begins in October, and if it is to be judged by the reputation and standing of the men by whom it is to be controlled and run, it is but fair to conclude that thorough work will be done.

The school has no connection with any other institution of learning and it has a fair opportunity to show what can be done under such circumstances. If it works up to the promise in the announcement, it will be one of our first-class schools so far as work is concerned. Some one has asked, "Does Indiana need another dental college?" Perhaps it does, if Ohio needs and has five, Michigan two, Pennsylvania four and Illinois four, five, six, seven or more, Indiana ought to sustain two. If the new college works up closely to its announcement, probably its success will be equal to the desire of its promoters.

